# Bacterial bile duct colonization in perihilar cholangiocarcinoma and its clinical significance

**DOI:** 10.1038/s41598-021-82378-y

**Published:** 2021-02-03

**Authors:** Jan Bednarsch, Zoltan Czigany, Lara Rosaline Heij, Tom Luedde, Ronald van Dam, Sven Arke Lang, Tom Florian Ulmer, Mathias Walter Hornef, Ulf Peter Neumann

**Affiliations:** 1 Department of Surgery and Transplantation, University Hospital RWTH Aachen, Pauwelsstrasse 30, 52074 Aachen, Germany; 2 Institute of Pathology, University Hospital RWTH Aachen, Aachen, Germany; 3 Department of Gastroenterology, Hepatology and Infectious Diseases, University Hospital Heinrich Heine University Duesseldorf, Düsseldorf, Germany; 4 Department of Surgery, Maastricht University Medical Center (MUMC), Maastricht, The Netherlands; 5 Institute of Medical Microbiology, University Hospital RWTH Aachen, Aachen, Germany

**Keywords:** Hepatology, Liver cancer, Applied microbiology, Bacteriology

## Abstract

Abdominal infections including cholangitis represent a major problem in patients with perihilar cholangiocarcinoma (pCCA). Thus, we investigated bacterial colonization of the bile ducts and determined its impact on postoperative outcome focusing on abdominal infections. A cohort of 95 pCCA patients who underwent surgery between 2010 and 2019 with available intraoperative microbial bile cultures were analyzed regarding bile duct colonization and postoperative abdominal infection by group comparisons and logistic regressions. 84.2% (80/95) showed bacterial colonization of the bile ducts and 54.7% (52/95) developed postoperative abdominal infections. *Enterococcus faecalis* (38.8%, 31/80), *Enterococcus faecium* (32.5%, 26/80), *Enterobacter cloacae* (16.3%, 13/80) and *Escherichia coli* (11.3%, 9/80) were the most common bacteria colonizing the bile ducts and *Enterococcus faecium* (71.2%, 37/52), *Enterococcus faecalis* (30.8%, 16/52), *Enterobacter cloacae* (25.0%, 13/52) and *Escherichia coli* (19.2%, 10/52) the most common causes of postoperative abdominal infection. Further, reduced susceptibility to perioperative antibiotic prophylaxis (OR = 10.10, *p* = .007) was identified as independent predictor of postoperative abdominal infection. Bacterial colonization is common in pCCA patients and reduced susceptibility of the bacteria to the intraoperative antibiotic prophylaxis is an independent predictor of postoperative abdominal infections. Adapting antibiotic prophylaxis might therefore have the potential to improve surgical outcome pCCA patients.

## Introduction

Perihilar cholangiocarcinoma (pCCA) is the second most common primary liver tumor and associated with a dismal overall oncological prognosis and high perioperative morbidity and mortality^1,2^. Extensive liver resection with radical lymphadenectomy and vascular resection is the current gold standard for the treatment of patients with resectable disease showing encouraging survival rates up to 60% after 5 years in various selected cohorts^3–6^. In contrast, palliative treatment modalities such as systemic chemotherapy result in a significantly inferior oncological outcome^7^. However, due to its direct proximity to major vascular structures of the liver hilus, surgical therapy of pCCA patients remains challenging and often displays significant perioperative mortality rates exceeding 10%^6,8–10^.

Given the extraordinarily high perioperative morbidity and mortality in these individuals, preoperative endoscopic biliary drainage (EBD) or percutaneous biliary drainage (PBD) are commonly used to release the pressure in the bile duct system, improve the liver function and promote postoperative functional recovery^11,12^. Due to preexisting conditions such as bile duct dilatation and reduced bile flow as well as the increased risk of bacterial contamination during the above-mentioned preoperative procedures, bacterial cholangitis develops in a significant proportion of pCCA patients and has been identified as an independent preoperative predictor of increased perioperative mortality^13–15^. Despite its potentially major impact on the perioperative outcome, the incidence and pattern of bacterial bile duct colonization have not been investigated so far. Bacterial bile duct colonization and cholangitis might also lead to postoperative abdominal infections with the risk to cause bacterial sepsis with often fulminant or even fatal complications and a reduced functional recovery of the remnant liver^16^. Previous reports have identified abdominal infections as a major source of adverse clinical outcomes, playing a key role in the development of postoperative complications, post-hepatectomy liver failure, and vascular complications^6,8–10^.

Thus, we here aimed to investigate bacterial bile duct colonization in a large European cohort of pCCA patients and determine its impact on the postoperative outcome focusing on abdominal infections.

## Material and methods

### Patients

Between 2010 and 2019, all surgically treated patients with localized pCCA at the University Hospital RWTH Aachen (UH-RWTH) with available information on bile duct colonization from intraoperative sampling were included in this study. Informed consent was obtained from all study participants. The study was conducted in accordance with the requirements of the Institutional Review Board of the RWTH-Aachen University (EK 114/20), the current version of the Declaration of Helsinki, and the good clinical practice guidelines (ICH-GCP). All clinical data were prospectively collected and entered in an institutional database.

### Staging and surgical technique

All pCCA patients who were referred to our institution for surgical treatment underwent a detailed clinical work-up as previously described^9,10^. In brief, the decision for surgery as primary treatment and the specific surgical procedure was made by an experienced hepatobiliary surgeon and approved by the local interdisciplinary tumor board in all cases. The specific tumor anatomy was assessed by endoscopic retrograde cholangiopancreatography (ERCP) or magnetic resonance cholangiopancreatography (MRCP), while the vascular anatomy at the liver hilum was assessed by multiphase computed tomography (CT). Our preoperative work-up included unilateral stenting strategy as standard of care to relieve the future liver remnant (FLR) from cholestasis and bilateral stenting in cases with persisting cholangitis. Endoscopic biliary drainage (EBD) was generally preferred over percutaneous biliary drainage (PBD). In patients with insufficient FLR (< 40%) scheduled for right-sided hepatectomy, a right portal vein embolization (PVE) was conducted 2–4 weeks before surgery. Prior to skin incision, the patients received a single shot antibiotic prophylaxis with cefuroxime and metronidazole in accordance with common guidelines for the prevention of surgical site infections^16^.

The surgical procedure was carried out as previously described by Neuhaus et al.^4,6,9,10,17^. Briefly, a “no-touch” hilar en-bloc resection approach, as defined by liver resection with mandatory portal vein reconstruction, was carried out in all cases and additional arterial resection or the concomitant resection of the pancreatic head (hepatoduodenopancreatectomy) on demand (Table 1). The actual parenchymal transection was carried out using the Cavitron Ultrasonic Surgical Aspirator (CUSA) with low central venous pressure. The extrahepatic bile ducts and the bile duct bifurcation is removed en-bloc with the liver specimen during parenchymal transection exposing the bile ducts to the FLR at the resection surface. A swab sample of the bile ducts for microbiological testing is immediately taken by the attending surgeon. Afterwards, biliary reconstruction was achieved by an end-to-side hepaticojejunostomy comprising an open hand-sewn anastomosis technique followed by a Roux-en-Y reconstruction. All surgical specimens were evaluated by an experienced board-certified staff pathologist regarding pathological characteristics according to current national guidelines, WHO- and UICC-classifications.

**Table 1:** Clinical and microbiological characteristics (n = 95).

**Demographics**	
Gender, m/f (%)	71 (74.7)/24 (25.3)
Age (years)	69 (56–73)
BMI (kg/m^2^)	25 (23–29)
Portal vein embolization, n (%)	43 (45.3)
ASA, n (%)	
I	5 (5.3)
II	36 (37.9)
III	51 (53.7)
IV	3 (3.2)
V	0
Bismuth type, n (%)	
I	4 (4.2)
II	10 (10.5)
IIIa	29 (30.5)
IIIb	19 (20.0)
IV	33 (34.7)
EBD, n (%)	82 (86.3)
PBD, n (%)	26 (27.4)
Preoperative cholangitis, n (%)	32 (33.7)
**Preoperative liver function**	
Albumin (g/dl)	36.9 (29.8–41.0)
AST (U/l)	45 (34–64)
ALT (U/l)	60 (35–99)
GGT (U/l)	380 (186–753)
Total bilirubin (mg/dl)	1.14 (0.62–2.43)
Platelet count (/nl)	298 (227–392)
Alkaline Phosphatase (U/l)	245 (159–396)
Prothrombine time (%)	95 (81–103)
INR	1.04 (0.97–1.13)
Hemoglobin (g/dl)	12.3 (11.3–13.3)
CRP (mg/dl)	16 (8–37)
**General operative data**	
Laparoscopic resection, n (%)	0
Operative time (min)	439 (360–505)
Operative procedure, n (%)	
Left hepatectomy	8 (8.4)
Extended left hepatectomy	26 (27.4)
Left trisectionectomy	4 (4.2)
Right hepatectomy	13 (13.7)
Extended right hepatectomy	17 (17.9)
Right trisectionectomy	26 (27.4)
Concomitant pancreatic resection, n (%)	7 (7.4)
Portal vein reconstruction, n (%)	95 (100)
Arterial resection, n (%)	5 (5.3)
Intraoperative blood transfusion (units)	0 (0–2)
Intraoperative FFP (units)	3 (0–4)
**Microbiological data**	
Preoperative antibiotics, n (%)	42 (44.2)
Antibiotic type, n (%)	
Ciprofloxacin	28 (66.7)
Piperacillin/tazobactam	6 (14.3)
Others	8 (19.0)
Perioperative prophylaxis, n (%)	95 (100.0)
Prophylaxis type, n (%)	
Cefuroxime/Metronidazole	95 (100.0)
Bacterial bile duct colonization, n (%)	80 (84.2)
Most common bacteria, n (%)	
* Enterococcus faecalis*	31 (38.8)
* Enterococcus faecium*	26 (32.5)
* Enterobacter cloacae*	13 (16.3)
* Escherichia coli*	9 (11.3)
* Klebsiella pneumoniae*	6 (7.5)
* Staphylococcus epidermidis*	6 (7.5)
* Klebsiella oxytoca*	5 (6.3)
* Hafnia alvei*	4 (5.0)
*Citrobacter freundii*	4 (5.0)
Susceptibility to preoperative antibiotics, n (%)	8 (22.2)
Susceptibility to applied perioperative antibiotics, n (%)	10 (12.5)
Postoperative abdominal infections, n (%)	52 (54.7)
Other postoperative infections, n (%)	18 (18.9)
Abdominal infections, bacteria, n (%)	
* Enterococcus faecium*	37 (71.2)
* Enterococcus faecalis*	16 (30.8)
* Enterobacter cloacae*	13 (25.0)
* Escherichia coli*	10 (19.2)
* Klebsiella pneumoniae*	9 (17.3)
* Staphylococcus epidermidis*	5 (9.6)
* Klebsiella oxytoca*	3 (5.8)
* Serratia marcescens*	3 (5.8)
**General postoperative data**	
Intensive care, days	2 (1–4)
Hospitalization, days	20 (13–40)
Postoperative complications, n (%)	
Clavien–Dindo ≤ IIIa	47 (49.5)
Clavien–Dindo ≥ IIIb	48 (50.5)
30-day mortality, n (%)	14 (14.7)
90-day mortality, n (%)	17 (17.9)
In-hospital mortality, n (%)	18 (18.9)

Data presented as median and interquartile range if not noted otherwise.

*ALT* alanine aminotransferase, *ASA* American society of anesthesiologists classification, *AST* aspartate aminotransferase, *BMI* body mass index, *CRP* c-reactive protein, *EBD* endoscopic biliary drainage, *FFP* fresh frozen plasma, *GGT* gamma glutamyltransferase, *INR* international normalized ratio, *PBD* percutaneous biliary drainage, *PVE* portal vein embolization.

### Microbiological analysis

Intraoperative bile samples were cultured in liquid media and on routine solid growth media for 18–48 h. Liquid cultures with signs of bacterial growth were subcultured on solid growth media. Bacterial isolates were identified by Maldi TOF mass spectrometric analysis (MALDI Biotyper, Bruker, Bremen, Germany) and antibiotic resistance was determined using an automated microdilution method (Vitek 2, bioMerieux, Nurtingen, Germany).

### Statistical analysis

Data derived from categorical data were presented in the form of numbers and percentages and tested for statistical difference using the chi-squared test, linear-by-linear association or fisher’s exact test in accordance to scale and number count. Continuous variables were presented as median and interquartile range and compared by the Mann–Whitney-U-Test. The associations of abdominal infections with clinical characteristics were assessed using univariate and multivariable logistic regression analyses in a forward selection model. The level of significance was set to *p* < 0.05 and *p* values were given for two-sided testing. Analyses were conducted using SPSS Statistics 24 (IBM Corp., Armonk, NY, USA).

## Results

### Patient cohort

A total of 127 patients underwent curative-intent surgery for pCCA at our institution between 2010 and 2019. As intraoperative bile sampling was not performed in every patient, only 95 individuals were included in the statistical analysis. This cohort consisted of 71 men (74.7%) and 24 women (31.5%) with a median age of 69 (range 56–73) years and median body mass index (BMI) of 25 (range 23–29). The majority of tumors were classified as Bismuth Type III or IV (85.3%, 81/95) and the majority of patients as ASA III or higher (56.9%, 54/95). Preoperative cholangitis was observed in 33.7% (32/95) of the cohort. EBD was performed in 86.3% (82/95) and PBD in 27.4% (26/95) in of the cases prior to surgery. Mandatory portal venous resection and reconstruction was carried out in all patients (95/95), while additional arterial reconstruction was conducted in 5.3% (5/95) and the concomitant resection of the pancreatic head in 7.4% (7/95) of the cases. Major morbidity defined as complications ≥ Clavien-Dindo IIIb was observed in 50.5% (48/95) and 30-day mortality in 14.7% (14/95) of the patients. The 90-day mortality and in-hospital mortality were was 17.9% (17/95) and 18.9% (18/95) in the study cohort with available intraoperative bile cultures (n = 95), while the in-hospital mortality was 15.7% (20/127) in the overall patient cohort (n = 127). More demographic and clinical characteristics of the cohort are presented in Table 1.

### Bacterial bile duct colonization and bacterial isolates of postoperative abdominal infections

Prior to surgery, 44.2% (45/95) of the patients received antibiotics as treatment or prophylaxis for cholangitis with ciprofloxacin representing the most frequently administered agent (66.7%, 28/45). Bacterial bile duct colonization as determined by intraoperative sampling was present in the vast majority of patients (84.5%, 80/95). The most commonly detected bacterial species were *Enterococcus faecalis* (38.8%, 31/80), *Enterococcus faecium* (32.5%, 26/80), *Enterobacter cloacae* (16.3%, 13/80), *Escherichia coli* (11.3%, 9/80), *Klebsiella pneumoniae* (7.5%, 6/80), *Staphylococcus epidermidis* (7.5%, 6/80), *Klebsiella oxytoca* (6.3%, 5/80), Hafnia *alvei* (5.0%, 4/80) and *Citrobacter freundii* (5.0%, 4/80). Notably, only a minor fraction of the bacterial isolates was susceptible to the preoperatively administered antibiotic (22.2%, 8/36) and to our standard perioperative antibiotic prophylaxis (12.5%,10/80) (Table 1).

Microbiologically confirmed postoperative abdominal infections were observed in 54.7% (52/95) with *Enterococcus faecium* (71.2%, 37/52), *Enterococcus faecalis* (30.8%, 16/52), *Enterobacter cloacae* (25.0%, 13/52), *Escherichia coli* (19.2%, 10/52), *Klebsiella pneumoniae* (17.3%, 9/52), *Staphylococcus epidermidis* (9.6%, 5/52), *Klebsiella oxytoca* (5.8%, 3/52) and *Serratia marcescens* (5.8%, 3/52) being the most commonly identified causative agents (Table 1). We further calculated a bile duct origin rate—defined as the presence of a specific bacterial species in the bile duct prior to surgery associated with a postoperative abdominal infection—as 94.5% (18/19) for *Enterococcus faecium*, 75.0% (3/4) for *Klebsiella pneumoniae*, 71.4% (5/7) for *Escherichia coli*, 61.1% (11/18) for *Enterococcus faecalis* and 55.6% (5/9) *Enterobacter cloacae* (Table 6). More details regarding bacterial colonization and postoperative abdominal infections also by less frequently observed bacterial isolates are outlined in Tables 1 and 6.

### Association of bacterial colonization with clinical parameters

Next, we performed a group comparison between patients with (n = 80) and without bacterial bile duct colonization (n = 15) at the time of surgery. No difference regarding gender (*p* = 0.247), age (*p* = 0.846) and BMI (*p* = 0.988) were observed. Patients with bacterial colonization had a significantly higher rate of prior EBD (91.3%, 73/80) compared to patients without bacterial colonization (60.0%, 9/15, *p* = 0.001). Also, preoperative cholangitis occurred more frequently in patients with colonized bile ducts (37.5%, 30/80) compared to patients without (13.3%, 2/15) but this difference did not reach statistical significance (*p* = 0.069). No difference was detected in the use of preoperative antibiotics between both groups (45.0% (36/80) vs. 40.0% (6/15), *p* = 0.720). While postoperative complications were observed with similar frequency (50.0% (40/80) vs. 53.3% (8/15), *p* = 0.813), a higher but statistically non-significant rate of 30-day mortality was observed in patients with bacterial colonization (16.3%, 13/80) in contrast to patients without bacterial colonization (6.7%, 1/15, *p* = 0.337). More details are shown in Table 2.

**Table 2:** Group comparison between patients with and without bacterial bile duct colonization.

	Bacterial colonization (n = 80)	No bacterial colonization (n = 15)	*p* value
Gender, m/f (%)	58 (72.5)/22 (27.5)	13 (86.7)/2 (13.3)	.247
Age (years)	69 (56–73)	67 (55–73)	.846
BMI (kg/m^2^)	25 (22–29)	25 (23–27)	.988
Portal vein embolization, n (%)	35 (43.8)	8 (53.3)	.494
ASA, n (%)			.426
I	5 (6.3)	0	
II	28 (35.0)	8 (53.3)	
III	44 (55.0)	7 (46.7)	
IV	3 (3.8)	0	
V	0	0	
Bismuth type, n (%)			.095
I	2 (2.5)	2 (13.3)	
II	8 (10.0)	2 (13.3)	
IIIa	22 (27.5)	7 (46.7)	
IIIb	17 (21.3)	2 (13.3)	
IV	31 (38.8)	2 (13.3)	
EBD, n (%)	73 (91.3)	9 (60.0)	**.001**
PBD, n (%)	23 (28.8)	3 (20.0)	.485
Preoperative cholangitis, n (%)	30 (37.5)	2 (13.3)	.069
Preoperative antibiotics, n (%)	36 (45.0)	6 (40.0)	.720
Antibiotic type, n (%)			.990
Ciprofloxacin	23 (63.9)	5 (83.3)	
Piperacillin/tazobactam	5 (13.9)	1 (16.7)	
Others	8 (22.2)	0	
Bacterial bile duct colonization, n (%)	80 (100)	0	
Most common bacteria, n (%)		n. a	
* Enterococcus faecalis*	31 (38.8)		
* Enterococcus faecium*	26 (32.5)		
* Enterobacter cloacae*	13 (16.3)		
* Escherichia coli*	9 (11.3)		
* Klebsiella pneumoniae*	6 (7.5)		
* Staphylococcus epidermidis*	6 (7.5)		
* Klebsiella oxytoca*	5 (6.3)		
* Hafnia alvei*	4 (5.0)		
* Citrobacter freundii*	4 (5.0)		
Susceptibility to preoperative antibiotics, n (%)	8 (25.0)	n. a	
Susceptibility to applied perioperative antibiotics, n (%)	10 (12.5)	n. a	
Postoperative abdominal infections, n (%)	45 (56.3)	7 (46.7)	.494
Other postoperative infections, n (%)	17 (21.3)	1 (14.3)	.186
Abdominal infections bacteria, n (%)			
* Enterococcus faecium*	32 (71.1)	5 (71.4)	.986
* Enterococcus faecalis*	12 (26.7)	4 (57.1)	.104
* Enterobacter cloacae*	12 (26.7)	1 (14.3)	.482
* Escherichia coli*	9 (20.0)	1 (14.3)	.721
* Klebsiella pneumoniae*	7 (15.6)	2 (28.6)	.397
* Staphylococcus epidermidis*	4 (8.9)	1 (14.3)	.652
* Klebsiella oxytoca*	3 (6.7)	0	.482
* Serratia marcescens*	1 (2.2)	2 (28.6)	**.005**
Intensive care, days	2 (1–9)	2 (1–4)	.715
Hospitalization, days	22 (13–41)	18 (21–31)	.345
Postoperative complications, n (%)			.813
Clavien–Dindo ≤ IIIa	40 (50.0)	7 (46.7)	
Clavien–Dindo ≥ IIIb	40 (50.0)	8 (53.3)	
30-day mortality, n (%)	13 (16.3)	1 (6.7)	.337

The various agents described here as “Preoperative antibiotics” describe the preoperative administration and do not refer to perioperative antimicrobial prophylaxis. Data presented as median and interquartile range if not noted otherwise. Bold indicates statistical significance (*p* < 0.05).

*ASA* American society of anesthesiologists classification, *BMI* body mass index, *EBD* endoscopic biliary drainage, *PBD* percutaneous biliary drainage, *PVE* portal vein embolization.

### Association of postoperative abdominal infections with clinical characteristics

Another group comparison was carried out between patients with (n = 52) and without (n = 43) abdominal infections. Similarly, no difference regarding gender (*p* = 0.067), age (*p* = 0.662) and BMI (*p* = 0.593) was observed. No association was detected with preoperative cholangitis (*p* = 0.822), the utilization of preoperative antibiotics (*p* = 0.675) or bacterial bile duct colonization (*p* = 0.494) as well as these bacteria's susceptibility to preoperatively administered antibiotics (*p* = 0.588). In contrast, susceptibility of the identified bacteria to the standard perioperative antibiotic regimen was more common in patients without postoperative abdominal infections (22.9% (8/43) vs. 4.4% (2/52), *p* = 0.013). Of note, other postoperative infections (pneumonia etc.) were more frequently observed in patients who already displayed abdominal infections (7.0% (3/43) vs. 28.8% (15/52), *p* = 0.007). Intensive care (*p* = 0.001) and hospital stay (*p* = 0.001) was longer and major postoperative complications were more common (30.2% (13/43) vs. 67.3% (35/52), *p* = 0.001) in patients with abdominal infections. Perioperative mortality was likewise higher in patients with abdominal infections (9.4% (4/43) vs.19.2% (10/52) but this difference did not reach statistical significance (*p* = 0.174). More details are presented in Table 3.

**Table 3:** Group comparison between patients with and without postoperative abdominal infection.

	Abdominal infection (n = 52)	No abdominal infection (n = 43)	*p* value
Gender, m/f (%)	35 (67.3)/17 (32.7)	36 (83.7)/7 (16.3)	.067
Age (years)	69 (57–74)	65 (56–72)	.662
BMI (kg/m^2^)	25 (23–29)	26 (23–30)	.593
Portal vein embolization, n (%)	31 (59.6)	12 (27.9)	**.002**
ASA, n (%)			.872
I	3 (5.8)	2 (4.7)	
II	21 (40.4)	15 (34.9)	
III	26 (50.0)	25 (58.1)	
IV	2 (3.8)	1 (2.3)	
V	0	0	
Bismuth type, n (%)			**.001**
I	2 (3.8)	2 (4.7)	
II	2 (3.8)	8 (18.6)	
IIIa	23 (44.2)	6 (14.0)	
IIIb	4 (7.7)	15 (34.9)	
IV	21 (40.4)	12 (27.9)	
EBD, n (%)	45 (86.5)	37 (86.0)	.945
PBD, n (%)	16 (30.9)	10 (23.3)	.414
Preoperative cholangitis, n (%)	17 (32.7)	15 (34.9)	.822
Preoperative antibiotics, n (%)	24 (46.2)	18 (41.9)	.675
Antibiotic type, n (%)			
Ciprofloxacin	18 (75.0)	10 (55.6)	
Piperacillin/tazobactam	2 (8.3)	4 (22.2)	
Others	4 (16.7)	4 (22.2)	
Bacterial bile duct colonization, n (%)	45 (86.5)	35 (81.4)	.494
Most common bacteria, n (%)			
* Enterococcus faecalis*	18 (40.0)	13 (37.1)	.795
* Enterococcus faecium*	19 (42.2)	7 (20.0)	**.035**
* Enterobacter cloacae*	9 (20.0)	4 (11.4)	.303
* Escherichia coli*	7 (15.6)	2 (5.7)	.167
* Klebsiella pneumoniae*	4 (8.9)	2 (5.7)	.593
* Staphylococcus epidermidis*	3 (6.7)	3 (8.6)	.741
* Klebsiella oxytoca*	2 (4.4)	3 (8.6)	.449
* Hafnia alvei*	1 (2.2)	1 (2.9)	.438
* Citrobacter freundii*	2 (4.4)	2 (5.7)	.796
Susceptibility to preoperative antibiotics, n (%)	4 (16.7)	4 (26.6)	.588
Susceptibility to applied perioperative antibiotics, n (%)	2 (4.4)	8 (22.9)	**.013**
Postoperative abdominal infections, n (%)	52 (100)	0	
Other postoperative infections, n (%)	15 (28.8)	3 (7.0)	**.007**
Abdominal infections bacteria, n (%)		n. a	
* Enterococcus faecium*	37 (71.2)		
* Enterococcus faecalis*	16 (30.8)		
* Enterobacter cloacae*	13 (25.0)		
* Escherichia coli*	10 (19.2)		
* Klebsiella pneumoniae*	9 (17.3)		
* Staphylococcus epidermidis*	5 (9.6)		
* Klebsiella oxytoca*	3 (5.8)		
* Serratia marcescens*	3 (5.8)		
Intensive care, days	3 (2–3)	1 (1–2)	**.001**
Hospitalization, days	32 (21–52)	13 (11–19)	**.001**
Postoperative complications, n (%)			
Clavien–Dindo ≤ IIIa	17 (32.7)	30 (69.8)	**.001**
Clavien–Dindo ≥ IIIb	35 (67.3)	13 (30.2)	
30-day mortality, n (%)	10 (19.2)	4 (9.4)	.174

The various agents described here as “Preoperative antibiotics” describe the preoperative administration and do not refer to perioperative antimicrobial prophylaxis. Data presented as median and interquartile range if not noted otherwise. Bold indicates statistical significance (*p* < 0.05).

*ASA* American society of anesthesiologists classification, *BMI* body mass index, *EBD* endoscopic biliary drainage, *PBD* percutaneous biliary drainage, *PVE* portal vein embolization.

### Univariate and multivariable analysis of postoperative abdominal infections

To determine predictors of abdominal infection logistic regression analyses were carried out. In univariate analysis, PVE (OR = 3.81, *p* = 0.002), right-sided hepatectomy (OR = 3.62, *p* = 0.004), intraoperative blood transfusions (OR = 3.69, *p* = 0.003), bile duct colonization by *Enterococcus faecium* (OR = 2.96, *p* = 0.031) and reduced susceptibility to perioperative antibiotic prophylaxis (OR = 6.37, *p* = 0.025) were associated with postoperative abdominal infections. All variables with a *p* value < 0.1 were subsequently analyzed in a multivariable model. Here, PVE (OR = 3.59, *p* = 0.015) and reduced susceptibility to the antibiotics administered as perioperative prophylaxis (OR = 10.10, *p* = 0.007) were identified as independent predictors of postoperative infection (Table 4).

**Table 4:** Univariate and multivariable analysis of postoperative abdominal infections.

	Univariate analysis		Multivariate analysis	
	OR (95% CI)	*p* value	HR (95% CI)	*p* value
Sex (male = 1)	2.50 (.92–6.76)	.072	Excluded	
Age (≤ 65 years = 1)	1.98 (.87–4.53)	.106		
BMI (≤ 25 kg/m^2^ = 1)	.67 (.30–1.51)	.329		
PVE (no = 1)	3.81 (1.60–9.07)	**.002**	3.59 (1.29–10.03)	**.015**
ASA (I/II = 1)	.76 (.34–1.73)	.517		
Bismuth type (IV = 1)	1.75 (.74–4.16)	.206		
EBD (no = 1)	1.04 (.32–3.37)	.945		
PBD (no = 1)	1.47 (.58–3.68)	.415		
Preoperative cholangitis (no = 1)	.91 (.39–2.13)	.822		
Type of hepatectomy				
Left-sided hepatectomy	1			
Right-sided hepatectomy	3.62 (1.52–8.60)	**.004**	Excluded	
Blood transfusion (no = 1)	3.69 (1.57–8.71)	**.003**	Excluded	
FFP transfusion (no = 1)	1.79 (.78–4.12)	.171		
Preoperative antibiotics (no = 1)	1.19 (.53–2.69)	.675		
Antibiotic type (Ciprofloxacin = 1)	.42 (.11–1.55)	.190		
Bacterial bile duct colonization (no = 1)	1.47 (.49–4.44)	.495		
*Enterococcus faecalis* (no = 1)	1.22 (.51–2.90)	.650		
*Enterococcus faecium* (no = 1)	2.96 (1.10–7.95)	**.031**	Excluded	
*Enterobacter cloacae* (no = 1)	2.04 (.58–7.16)	.265		
*Escherichia coli* (no = 1)	2.56 (.26–25.78)	.424		
*Klebsiella pneumoniae* (no = 1)	1.71 (.30–9.81)	.548		
*Staphylococcus epidermidis* (no = 1)	.82 (.16–4.27)	.810		
*Klebsiella oxytoca* (no = 1)	.53 (.09–3.35)	.502		
*Hafnia alvei* (no = 1)	2.57 (.26–25.66)	.421		
*Citrobacter freundii* (no = 1)	.82 (.11–6.01)	.846		
Susceptibility to preoperative antibiotics (no = 1)	1.55 (.32–7.50)	.589		
Susceptibility to applied perioperative antibiotics (no = 1)	6.37 (1.26–32.27)	**.025**	10.10 (1.90–53.67)	**.007**

Various parameters are associated with postoperative abdominal infections. Bold indicates statistical significance (*p* < 0.05).

*ASA* American society of anesthesiologists classification, *BMI* body mass index, *EBD* endoscopic biliary drainage, *PBD* percutaneous biliary drainage, *PVE* portal vein embolization.

### Antibiotic susceptibility of preoperatively colonizing bacteria

Finally, we compared the antibiotic susceptibility of the bacterial bile duct isolates with the standard perioperative antibiotic regimen. While only 12.5% (10/80) of the bile duct colonizing bacterial isolates were susceptible to the standard perioperative antibiotic agent cefuroxime, 75.0% (60/80) were susceptible to a combination of ceftriaxone and vancomycin (Table 5).

**Table 5:** Susceptibility of the bile duct colonizing isolates to antibiotic agents.

Antibiotic agent	Most common bacteria^a^	All bacteria
Cefuroxime	5.9% (4/68)	12.5% (10/80)
Ceftriaxone	13.2% (9/68)	17.5% (14/80)
Ciprofloxacin	14.7% (10/68)	16.3% (13/80)
Ampicillin/sulbactam	30.9% (21/68)	28.8% (23/80)
Piperacillin/tazobactam	47.1% (32/68)	47.5% (38/80)
Vancomycin	50.0% (34/68)	43.8% (35/80)
Ampicillin/sulbactam + Ciprofloxacin	54.4% (37/68)	58.8% (47/80)
Piperacillin/tazobactam + Ciprofloxacin	57.4% (39/68)	61.3% (49/80)
Meropenem	64.7% (44/68)	68.8% (55/80)
Cefuroxime + Vancomycin	64.7% (44/68)	53.8% (43/80)
Ceftriaxone + Vancomycin	85.3% (58/68)	75.0% (60/80)
Meropenem + Vancomycin	98.5% (67/68)	97.5% (78/80)

^a^The most common bacteria which were found in the bile ducts were *Enterococcus faecium*, *Enterococcus faecalis*, *Enterobacter chlocae*, *Escherichia coli*, *Klebsiella pneumoniae*, *Staphylococcus epidermidis*, *Klebsiella oxytoca*, *Hafnia alvei* and *Citrobacter freundii.*

## Discussion

Radical surgery is the therapeutic mainstay in pCCA as it displays superior oncological outcome compared to palliative treatment modalities^7^. Therefore, improving the perioperative outcome of patients undergoing major hepatic resection for pCCA would significantly improve their overall outcome^1,2,6,8–10^. While preoperative cholangitis has previously been identified as an independent predictor of an adverse outcome, the incidence of bacterial bile duct colonization and its impact on the clinical management and outcome have not been determined^13,14^.

In the present study, we investigated the influence of bacterial bile duct colonization on the clinical outcome of patients with pCCA. Here we provide the results of a relatively large European cohort of pCCA patients and demonstrated that bacterial bile duct colonization occurs in the vast majority of patients. We also identified reduced susceptibility of the bacterial isolates to the administered perioperative antibiotic prophylaxis as an independent predictor of postoperative abdominal infections in the multivariate analysis. Based on these observations we speculate that a change in the perioperative antibiotic prophylaxis might significantly reduce postoperative abdominal infections and subsequently improve the overall clinical outcome in pCCA patients.

Bacterial cholangitis has been identified as major risk factor in patients with biliary tract disease such as malign and benign bile duct stenosis or choledocholithiasis among others^18–21^. However, the main research focus has been the choice of the empirical antibiotic therapy with respect to the common bacterial spectrum of acute cholangitis and not the exploration of bacterial bile duct colonization and its impact on the subsequent surgical intervention. Also, in comparison to our present analysis of pCCA patients, most previous reports have included a rather heterogeneous patient cohort including a broad range of different biliary tract diseases and clinical conditions^18–21^. The microbiological findings of our patients are in line with these heterogeneous cohorts with *Enterococcus spp.* being the most relevant gram-positive bacterium and *Enterobacter cloacae*, *Escherichia coli* and *Klebsiella pneumoniae* representing the most common gram-negative bacterial biliary isolates^18–21^. Interestingly, gram-negative bacteria appeared clinically more relevant in these previous reports as they showed a higher likelihood to be also detected in blood cultures^18,20^. In our patient cohort, gram-positive bacteria, particularly *Enterococcus faecium*, seemed to have a major clinical importance since preoperative colonization with *Enterococcus faecium* was associated with a significantly increased risk of postoperative abdominal infection in our univariate analysis (Table 4). Also, gram-positive bacteria were more commonly detected as causative agents of postoperative abdominal infections in these patients with *Enterococcus faecium* being isolated in 71.2% of all abdominal infections compared to *Enterobacter cloacae* as the most frequent gram-negative bacterium in only 25.0% of all abdominal infections (Table 1). These results demonstrate notable differences in the clinical significance of bacterial isolates in cholangitis in general and postoperative abdominal infections after surgical resection of pCCA in particular.

Of note, the only available comparable studies to our report, though on the basis of Asian patients, heterogeneous tumor entities and significantly different clinical management and objectives, have been published from the Nagoya group. Sugawara et al. have shown in a randomized-controlled that a two-day and four-day postoperative administration of antimicrobial prophylaxis results in the same clinical outcome^22^. As a secondary finding in this study, preoperative bile cultures were colonized mostly with *Enterococcus species*, *followed by Klebsiella species* and *Enterobacter species* which was similar to our findings and does further underline the importance of gram-positive bacteria in pCCA^22^. In another retrospective cohort study focusing on the role of preoperative biliary drainage, the same group observed an analogous microbial spectrum and indicated that postoperative infections might display the same bacteria as previously detected in preoperative bile samples^23^. Interestingly, preoperative biliary colonization was observed in 84% of all patients comparable with our data, but was also associated with an increased risk of postoperative infections which was not the case in our study^23^. However, the interesting findings of both studies are not transferable to our setting as they included a significant proportion of patients with gallbladder cancer, other malignancies and benign strictures, focused on preoperative bile samples instead of intraoperatively obtained swap samples and most importantly, conducted a different perioperative strategy regarding antibiotics among other differences in clinical management (e.g. use of nasobiliary drainage etc.)^22,23^. In the large retrospective cohort study of Sugawara et al., postoperative antibiotic prophylaxis was used for at least 3 days after operation, even in cases without preoperative bacterial colonization, while this approach was not conducted in our cohort^23^. Further, susceptibility of the cultivated bile samples to the intraoperatively applied antibiotics was not reported in this study^23^.

As already mentioned above, the vast majority of our patients presented with bacterial colonization of the bile ducts at the time of surgery (Table 1). The most likely explanation for this is the high number of endoscopic interventions in pCCA patients prior to definitive surgery to achieve biliary decompression of the FLR^11,12^. Biliary endoprostheses are a known risk factor for bacterial cholangitis and notability associated with a higher risk of *Enterococcus faecium* colonization^18,24,25^. This observation was confirmed in our analysis as a biliary plastic stent in situ placed by EBD was significantly more common in patients with bacterial colonization compared to patients without bacterial colonization at the time of surgery. However, we were not able to identify other differences in clinical characteristics between patients with and without bacterial colonization. In particular, while preoperative cholangitis showed a tendency to be more common in patients with bile duct colonization, we observed no difference in the preoperative antibiotic use between individuals with and without bacterial colonization.

Major surgery required for radical resection of pCCA is often associated with significant morbidity and increased perioperative mortality^26,27^. In our particular cohort the 30-day mortality was 14.7% and major morbidity defined as complications categorized ≥ IIIb according to the Clavien-Dindo scale were observed in more than half of the patients. Our comparative analysis of patients with and without postoperative abdominal infections showed significantly more major complications and a tendency for a higher mortality (Table 3). This observation confirms the importance of postoperative abdominal infections as a major cause for an adverse outcome.

Subsequently, we conducted a logistic regression analysis to identify independent predictors for postoperative abdominal infection and identified reduced susceptibility of the bacterial bile duct isolates to the perioperatively applied antibiotics as the most relevant predictor in our multivariable analysis (Table 4). During surgery for pCCA, the extrahepatic bile ducts are resected together with the hemi-liver to obtain clear tumor margins^6,8–10^. The biliary reconstruction is afterwards achieved by a hepaticojejunostomy^28^. Intraoperatively the abdominal cavity is inevitably contaminated by biliary secretion from the exposed major bile ducts of the resection surface. The results of our multivariable analysis support the hypothesis of a relevant association between the above-mentioned intraoperative exposure of the abdominal situs to bile fluid and postoperative abdominal infections. They may therefore have important implications for the selection of an adequate perioperative antibiotic prophylaxis. This is consistent with the elevated rate of persistence of *Enterococcus faecium* illustrated by the fact that out of 19 patients with postoperative abdominal infections caused by *Enterococcus faecium*, 18 patients (94.5%) also showed *Enterococcus faecium* in the intraoperatively collected bile cultures (Table 6). The close association of bile duct colonization at the timepoint of surgery with postoperative infections underlines the importance of intraoperative bile cultures to better adjust individual postoperative antibiotic treatment and to collect a larger amount of clinical data and eventually optimize future empirical prophylaxis on a larger populational level.

**Table 6:** Identified bacterial species causing the bile duct colonization or postoperative abdominal infections.

Specific bacteria	Bile duct isolates	Postoperative abdominal infection isolates	Bile duct origin rate
*Aeromonas caviae*	2.5% (2/80)	–	–
*Atopobium rimae*	–	1.9% (1/52)	–
*Bacillus pumilus*	1.3% (1/80)	–	–
*Bacteroides fragilis*	1.3% (1/80)	–	–
*Bacteroides pyogenes*	–	1.9% (1/52)	–
*Bacteroides thetaiotaomicron*	–	1.9% (1/52)	–
*Bacteroides uniformis*	–	1.9% (1/52)	–
*Bacteroides vulgatus*	–	1.9% (1/52)	–
*Citrobacter freundii*	5.0% (4/80)	1.9% (1/52)	50.0% (1/2)
*Clostridium perfringen*	–	1.9% (1/52)	–
*Enterobacter aerogenes*	–	1.9% (1/52)	–
*Enterobacter cloacae*	16.3% (13/80)	25.0% (13/52)	55.6% (5/9)
*Enterococcus casseliflavus*	1.3% (1/80)	–	–
*Enterococcus durans*	2.5% (2/80)	3.8% (2/52)	(0/0)
*Enterococcus faecalis*	38.8% (31/80)	30.8% (16/52)	61.1% (11/18)
*Enterococcus faecium*	32.5% (26/80)	71.2% (37/52)	94.5% (18/19)
*Escherichia coli*	11.3% (9/80)	19.2% (10/52)	71.4% (5/7)
*Hafnia alvei*	5.0% (4/80)	–	–
*Klebsiella ornithinolytica*	2.5% (2/80)	1.9% (1/52)	(0/1)
*Klebsiella oxytoca*	5.0% (4/80)	5.8% (3/52)	(0/2)
*Klebsiella pneumoniae*	7.5% (6/80)	17.3% (9/52)	75.0% (3/4)
*Klebsiella variicola*	1.3% (1/80)	–	–
*Lactobacillus fermentum*	–	3.8% (2/52)	–
*Lactobacillus paracasei*	–	1.9% (1/52)	–
*Morganella morgagnii*	–	3.8% (2/52)	–
*Orchobactrum anthropi*	3.8% (3/80)	–	–
*Pediococcus acidilactici*	1.3% (1/80)	–	–
*Pediococcus pentosaceus*	1.3% (1/80)	–	–
*Prevotella bivia*	1.3% (1/80)	1.9% (1/52)	(0/1)
*Prevotella melaninogenica*	–	3.8% (2/52)	–
*Proteus penneri*	1.3% (1/80)	–	–
*Proteus vulgaris*	2.5% (2/80)	–	–
*Provetella buccae*	–	3.8% (2/52)	–
*Pseudomonas aeruginosa*	3.8% (3/80)	3.8% (2/52)	100% (2/2)
*Serratia marcescens*	3.8% (3/80)	5.8% (3/52)	50.0% (1/2)
*Serratia rubidaea*	1.3% (1/80)	3.8% (2/52)	100% (1/1)
*Sphingomonas paucimobilis*	1.3% (1/80)	–	–
*Staphylococcus aureus*	3.8% (3/80)	3.8% (2/52)	(0/2)
*Staphylococcus epidermidis*	7.5% (6/80)	9.6% (5/52)	66.7% (2/3)
*Staphylococcus haemolyticus*	1.3% (1/80)	3.8% (2/52)	(0/0)
*Staphylococcus hominis*	1.3% (1/80)	–	–
*Stenotrophomonas maltophilia*	2.5% (2/80)	1.9% (1/52)	100% (1(1)
*Streptococcus anginosus*	5.0% (4/80)	5.8% (3/52)	(0/2)
*Streptococcus mitis*	1.3% (1/80)	1.9% (1/52)	(0/1)
*Streptococcus parasanguinis*	2.5% (2/80)	–	–
*Streptococcus peroris*	1.3% (1/80)	–	–
*Streptococcus sanguinis*	2.5% (2/80)	–	–

The percentage of specific bacteria of the bacterial isolates in the bile ducts and postoperative abdominal infections are shown. The bile duct origin rate is defined as the probability of a specific bacteria causing postoperative abdominal infection to already have colonized the bile ducts prior to surgery.

Perioperative antibiotic prophylaxis is an internationally accepted measure to reduce surgical site infections in patients undergoing abdominal surgery^16^. Our in-house policy during the study period comprised a single shot antibiotic prophylaxis with a second generation cephalosporine (cefuroxime) plus metronidazole in patients with pCCA in accordance with clinical standards^29^. Of note, only a small subset (12.5%) of the bile duct isolates was susceptible to the antibiotic prophylactic regimen. This reflects the fact that this antibiotic regimen has been selected to prevent surgical site infections in a general surgical population and not to address the specific and inevitable bile contamination of the abdominal cavity during pCCA surgery (Table 5). As the perioperatively administered antibiotics were the strongest predictor for postoperative abdominal infection in our cohort, we assessed the susceptibility of the bile duct isolates to various additional antibiotics. Here, vancomycin with 43.8% and meropenem with 68.8% were the most effective antibiotics with the combination of both covering 97.5% of all isolates (Table 5).

Our findings identify an important clinical factor with a potentially significant impact in the management of these high-risk patients and suggest a modification of the perioperatively applied antibiotics to improve postoperative outcome. While a combination of meropenem and vancomycin appears to cover the vast majority of all bacterial bile duct isolates, it has to be acknowledged that the application of two broad-spectrum antibiotics during surgery might facilitate the development of multidrug-resistant bacteria and can complicate the selection of antibiotics in patients presenting with septic complications in the early postoperative period. Considering this potential relevant adverse effect, our standard antibiotic prophylaxis was changed to a third generation cephalosporine (ceftriaxone) supplemented by vancomycin to cover *Enterococcus faecium* (observed in 80.0% of all bile duct isolates).

The duration of perioperative prophylaxis in pCCA is topic of an ongoing debate and typical regimes vary from a classical one-time prophylaxis during surgery to prolonged perioperative prophylaxis including the early postoperative period (e.g. 48 h)^22^. While there is clinical evidence from a randomized-controlled trial of the Nagoya group that there is no difference in outcome between a two-day and four-day postoperative prophylaxis, no systemic evaluation of a one-time prophylaxis versus a short (48 h) postoperative prophylaxis is currently available in the literature^22^. Unfortunately, this relevant question cannot be further explored with the help of our data as our strategy comprised a one-time prophylaxis during the complete study period. However, given the importance of the issue, clinical trials are warranted which should investigate the optimal duration of prophylaxis in this complex disease which may help to further improve surgical outcomes in pCCA patients.

Penetration of the antibiotic agent to the biliary lumen has historically been considered one of the major factors in the selection of antibiotics. Unfortunately, vancomycin’s ability to reach the bile fluid (as indicated by a relatively low bile to serum concentration ratio) is limited^30^. However, the current Tokyo Guidelines on antibacterial treatment for cholangitis and cholecystitis still recommend the use of vancomycin in case of *Enterococcus faecium* associated infections^31^. Moreover, there is some evidence that the secretion of antibiotics into bile fluid is altered by biliary obstruction, a condition frequently observed in pCCA patients despite preoperative drainage^31,32^. Also, the establishment of antibiotic concentrations within the abdominal cavity to reduce the spread of bile-derived bacteria to the abdominal lumen during the intervention might prevent abdominal infection even in the absence of a reduced bile duct colonization.

Similar to other retrospective clinical outcome studies, our analysis has a number of limitations, which should be considered. All patients of this study underwent surgery in a single center in accordance with the authors individual approach to pCCA and all data were obtained in a retrospective fashion. Although a multicenter approach may have increased the value of our analysis in terms of sample size, it may also introduce an increased heterogeneity in the clinical management and operative techniques. Unfortunately, as described in the methods section, intraoperative bile cultures were not available for all patients who underwent surgery for pCCA during the study period, which may have introduced a certain selection bias.

This study represents, to the best of our knowledge, the first European report investigating bacterial bile duct colonization and its influence on the postoperative outcome of patients undergoing curative-intent surgery for pCCA. Despite the aforementioned limitations, our analysis identified bacterial bile duct colonization as a common condition of patients undergoing surgery for pCCA and the reduced susceptibility to the intraoperatively administered antibiotic prophylaxis as an independent predictor for postoperative abdominal infection. As postoperative abdominal infections are a major cause of postoperative morbidity and mortality, a modified perioperative antibiotic prophylaxis might improve the operative outcome in patients with pCCA. Larger multicentric analyses and prospective controlled trials are needed to confirm and validate our findings.

## Data Availability

Available upon request. JB and UPN had full access to the data and act both as guarantor for the data.

## Abbreviations

ALT: Alanine aminotransferase
AP: Alkaline phosphatase
ASA: American society of anesthesiologists
AST: Aspartate aminotransferase
BMI: Body mass index
CCI: Comprehensive complication index
CI: Confidence interval
CRP: C reactive protein
CT: Computed tomography
CUSA: Cavitron ultrasonic surgical aspirator
EBD: Endoscopic biliary drainage
ERCP: Endoscopic retrograde cholangiopancreatography
FFP: Fresh frozen plasma
FLR: Future liver remnant
GGT: Gamma glutamyltransferase
INR: International normalized ratio
MRCP: Magnetic resonance cholangiopancreatography
PBD: Percutaneous biliary drainage
pCCA: Perihilar cholangiocarcinoma
RWTH: Rheinisch-Westfälische Technische Hochschule
UICC: Union for international cancer control
